# Comparison of acupuncture, moxibustion, and pharmacotherapy in improving diarrhea-predominant irritable bowel syndrome

**DOI:** 10.3389/fmicb.2025.1638930

**Published:** 2025-10-02

**Authors:** Peiqin Zhang, Yao Chen, Biyu Lai, Shuangshuang Wang, Dan Li, Runlin Wen, Yaping Duan, Dan Liu, Bo Li, Chang She

**Affiliations:** ^1^Changsha Hospital of Traditional Chinese Medicine (Changsha Eighth Hospital), Changsha, China; ^2^Institute of Rehabilitation and Health Care, Department of Rehabilitation and Traditional Chinese Medicine, Hunan Traditional Chinese Medical College, Zhuzhou, China

**Keywords:** irritable bowel syndrome, gut microbiota, moxibustion, acupuncture, colonic physiology

## Abstract

**Introduction:**

Acupuncture, moxibustion, and pharmacotherapy are widely used for diarrhea-predominant irritable bowel syndrome (IBS-D), but their comparative efficacy and microbial mechanisms remain unclear.

**Methods:**

We evaluated therapeutic outcomes in IBS-D rats through physiological and colonic indicators and characterized gut microbiota using 16S rDNA sequencing with network and modular analyses.

**Results:**

Acupuncture and moxibustion more effectively normalized stool form, diarrhea index, fecal water content, and fecal pellet counts compared to pharmacotherapy, which provided partial improvement with residual symptoms such as faster colonic transit. A total of 33 microbial taxa were identified as biomarkers, with distinct profiles associated with each intervention. Specifically, acupuncture was linked to increased levels of Pseudomonas and Turicibacter, moxibustion to RF39, and pharmacotherapy to Fusobacteriota, Blautia, and Clostridium_sensu_stricto_1. Furthermore, we also found that the ratio of Firmicutes to Bacteroidetes was a key factor in IBS-D pathogenesis. Key genera such as Muribaculum were modulated by the interventions to alleviate symptoms. Acupuncture and moxibustion restructured gut microbiota networks to improve connectivity and clustering, while pharmacotherapy resulted in a more heterogeneous network with a higher number of negative correlations with physiological parameters.

**Conclusion:**

These results demonstrated that acupuncture and moxibustion achieved superior therapeutic effects through distinct remodeling of microbial networks and host–microbe associations, providing mechanistic insight into microbiota-mediated IBS-D treatment.

## Introduction

Irritable bowel syndrome (IBS) is a chronic functional gastrointestinal disorder characterized by recurrent abdominal pain accompanied by altered bowel habits (Ford et al., 2020; Shaikh et al., 2023). Contemporary diagnostic frameworks classify IBS into diarrhea-predominant (IBS-D), constipation-predominant (IBS-C), mixed (IBS-M), and unclassified (IBS-U) subtypes based on stool pattern and consistency (Lacy et al., 2016; Longstreth et al., 2006; Su et al., 2023). A meta-analysis of 96 studies across 51 countries reported an overall IBS prevalence of 14%, with the diarrhea-predominant subtype accounting for 28% of cases (Bin Arif et al., 2024). In a multicenter cohort of 752 patients diagnosed by Rome IV criteria, individuals with IBS-D had significantly poorer disease-specific quality of life than those with IBS-C or IBS-M (mean [SD] IBS-QOL: 45.3 [23.0] vs. 52.3 [19.9] vs. 49.4 [22.0]; *p* = 0.005) and identified diarrhea as one of the most troublesome symptoms (Khasawneh et al., 2024). Dean et al. (2025) further demonstrated high susceptibility among IBS-D patients with a relative risk of 3.09 (95% CI = 2.41–3.97; *p* < 0.00001). Moreover, the annual management cost of IBS is estimated at approximately US$1 billion, highlighting a substantial healthcare burden and the need for cost-effective therapies (Bin Arif et al., 2024).

IBS-D emerges as the most prevalent subtype in epidemiological studies, posing considerable clinical management challenges due to its substantial disease burden and profound detrimental effects on patients’ quality of life (Chey et al., 2015; Holtmann et al., 2016; Longstreth et al., 2006). Management of IBS-D typically combines non-pharmacological strategies (dietary modification and bowel-directed behavioral therapies) with targeted pharmacotherapies (Nee and Lembo, 2021). Current treatments for IBS-D, such as antispasmodics, antidiarrheal agents, and probiotics, primarily address symptoms yet remain limited by frequent recurrence and treatment-related adverse effects, motivating comparative and mechanism-oriented investigations (Camilleri, 2021; Ford et al., 2020; Jakubczyk et al., 2020; Stojanov et al., 2020; Vich Vila et al., 2020).

Intestinal microbial dysbiosis is increasingly recognized as a relevant pathogenic factor for IBS-D and is characterized by compositional and functional imbalances of the gut microbiota (Margolis et al., 2021; Roager and Licht, 2018; Su et al., 2023). Traditional Chinese medicine (TCM) modalities, particularly acupuncture and moxibustion, have been investigated as complementary strategies for IBS-D (Bao et al., 2016; Fang et al., 2024; Savarino et al., 2022; Yaklai et al., 2021). Dai et al. (2022) compared the treatments of Western medicine, TCM prescription, and acupuncture across 11 randomized controlled trials, including 725 participants. They suggested that moxibustion demonstrated superior efficacy against IBS-D. However, direct comparisons of acupuncture, moxibustion, and conventional pharmacotherapy that integrate physiological readouts with microbiome profiling remain scarce.

Therefore, the present study compared acupuncture, moxibustion, and pharmacotherapy in a well-characterized rat model of IBS-D. We evaluated therapeutic effects using physiological and colonic characteristics and profiled fecal microbiota via 16S rDNA sequencing. Our objectives were to (1) delineate bacterial community changes and interaction networks that accompany treatment-associated improvements under each intervention and (2) contextualize these findings relative to conventional pharmacotherapy, clarifying whether and how microbiota remodeling tracks with observed therapeutic benefits.

## Materials and methods

### Induction of IBS-D model in rats

A total of 58 6- to 8-week-old male specific-pathogen-free (SPF) Sprague–Dawley rats (210 ± 10 g) were obtained from Hunan Slake Jingda Laboratory Animal Co., Ltd. (Production License: SCXK (Xiang) 2019-0004). Animals were housed under controlled conditions (humidity 50%–70%, temperature 20°C–25°C, and a 12 h light–dark cycle). After a 7-day acclimatization, 10 rats were designated as the normal control group (no model induction; saline enema), and 48 rats underwent IBS-D induction using a modified acetic acid-induced protocol (An et al., 2009).

On days 1 and 4, rats received a 1 ml 4% (v/v) acetic acid enema (glacial acetic acid in 0.01 moL/L PBS) via a paraffin oil-lubricated silicone tube inserted 6–8 cm from the anus, followed by manual anal compression for 60 s with the tail elevated at approximately 60°, and a 1 ml PBS colonic rinse. On days 2, 3, 5, 6, 7, and 8, model maintenance consisted of 1-h restraint in a cylindrical restrainer (restricting movement without compromising respiration) immediately followed by 5 min tail clamping at 3–5 cm from the tip. The induction period lasted a total of 8 days.

The success of the model was evaluated on day 8 using established criteria (Zhou et al., 2018), including changes in general condition, Bristol Stool Form Scale (BSFS) score, fecal water content (FWC) (measured by drying feces at 60 °C for 24 h), fecal pellet counts (FP), diarrhea index (DI), and colonic transit time (CTT). A total of 10 rats were excluded from the study due to modeling failure (*n* = 5), severe hematochezia (*n* = 1), or mortality (*n* = 4), leaving 38 successfully modeled rats. Modeling failure was predefined as no meaningful worsening in BSFS and DI relative to the normal controls according to prespecified thresholds. All procedures were approved by the Ethics Committee of Changsha Traditional Chinese Medicine Hospital/Changsha Eighth Hospital (Ethics Approval No.: 2019005) and followed the 2006 national Guidelines for the Care and Use of Laboratory Animals.

### Intervention methods

The 38 successfully modeled rats were randomized (computer-generated sequence) into four groups: model (M, *n* = 10), acupuncture (Ac, *n* = 9), moxibustion (Mx, *n* = 10), and pharmacotherapy (P, *n* = 9). The normal control group (C, *n* = 10) received no model induction and no therapeutic intervention. Interventions started the day after model evaluation and continued for 14 consecutive days. To maintain the IBS-D phenotype, all modeled groups (M, Ac, Mx, P) underwent daily 1-h restraint followed immediately by 5-min tail clamping; the control group (C) did not undergo either procedure. The specific interventions for each group were as follows:

(1) Control (C): No interventions were applied, and standard husbandry practices were followed.
(2) Model (M): Model maintenance only (daily restraint + tail clamping) without active therapy.
(3) Acupuncture (Ac): Sterile stainless-steel needles (0.25 × 25 mm) were inserted vertically to 5 mm at bilateral Zusanli (ST36) under aseptic conditions. Needles were retained for 15 min. Manual needle manipulation followed a standardized reinforcing-reducing (tonifying-reducing) technique with bidirectional rotation (about 180° in each direction) for 20 s at 5 min intervals throughout retention (Zhang, 2010).
(4) Moxibustion (Mx): Moxa stick stimulation (suspended moxibustion) was applied 10–15 cm above bilateral ST36 for 15 min per session. A custom surface-temperature monitoring setup with a 5 mm-thick ceramic thermal barrier (5 mm apertures) ensured consistent exposure (Zhang, 2010).
(5) Pharmacotherapy (P): pinaverium bromide was administered by oral gavage at 13.5 mg/kg/day (dose derived from human 150 mg/day using body surface area (BSA) conversion: 150 × 0.018 × 5), at a dosing volume of 10 ml/kg (suspension 1.35 mg/ml in sterile 0.9% NaCl).

### Specimen collection and analysis

At the end of the 14-day intervention, rats were evaluated for body mass, BSFS, FWC, DI, FP, and CTT. Rats were then anesthetized with 1.5% isoflurane (0.7 L/min) and humanely euthanized by anesthetic overdose (isoflurane ≥ 5% in oxygen until loss of corneal reflex and apnea, followed by cervical dislocation). The same overdose protocol was applied to all excluded animals (*n* = 10) in accordance with institutional guidelines. A 5 cm segment of the colon was collected 6 cm from the anus, longitudinally incised, and approximately 200 mg of fecal contents were stored at −80 °C for 16S rDNA sequencing of gut microbiota.

Body mass was measured on an electronic balance. The BSFS was scored according to the Bristol Stool Form Scale (Lewis and Heaton, 1997). FWC, FP, DI, and CTT were assessed as described previously (Lai et al., 2023). For stool-based readouts, rats were individually housed on liner paper for 4 h to collect fresh feces. FWC (%) = (Wet − Dry)/Wet ×100 after drying at 50 °C for 6 h. Loose stools were graded by diameter: Grade 1 < 1 cm; Grade 2 = 1 cm–< 2 cm; Grade 3 = 2 cm–3 cm; Grade 4 > 3 cm. DI = (Number of loose stools/Total stools) × Average loose-stool grade. CTT was determined by inserting a 3 mm glass bead 3 cm into the rectum under anesthesia and recording the time from awakening to bead expulsion.

### DNA extraction and 16S rDNA gene amplicon sequencing

Fecal samples (200 ± 5 mg) were collected and immediately stored at −80 °C. Total genomic DNA was extracted using the QIAamp Fast DNA Stool Mini Kit (Qiagen, Germany) and verified by 1% agarose gel electrophoresis. The V3-V4 regions of the bacterial 16S rRNA gene were amplified using barcoded primers (341F: 5′-CCTAYGGGRBGCASCAG-3′; 806R: 5′-GGACTACHVGGGTWTCTAAT-3′). PCR amplification was performed with TransStart FastPfu DNA Polymerase (TransGen Biotech, AP221-02) under the following conditions: 98 °C for 5 min; 25 cycles of 98 °C for 30 s, 53 °C for 30 s, 72 °C for 45 s, and 72 °C for 5 min. Amplicons were purified with VAHTSTM DNA Clean Beads (Vazyme, China) and quantified using the Quant-iT PicoGreen dsDNA Assay Kit (Invitrogen, USA). Equal amounts of purified amplicons were pooled, and paired-end 2 × 250 bp sequencing was performed on the Illumina MiSeq platform (MiSeq Reagent Kit v3) at Shanghai Personal Biotechnology Co., Ltd. (Shanghai, China).

### Microbial community analysis

Libraries were prepared using the TruSeq Nano DNA LT Library Prep Kit, followed by PCR amplification and purification with AMPure XP beads. Size selection was performed with 2% agarose gel electrophoresis. Library quality was assessed using an Agilent Bioanalyzer 2,100 (High Sensitivity DNA Kit), requiring a single peak without adapter dimers. Quantification was performed using the Quant-iT PicoGreen dsDNA Assay Kit (Promega QuantiFluor), with a minimum acceptable concentration of 2 nM. High-quality sequence analyses were performed using QIIME2 (v.2019.4) (Bolyen et al., 2018). Raw sequence data were demultiplexed with the demux plugin, primers trimmed with cutadapt (Martin, 2011), and sequences denoised, merged, and chimeras checked using the DADA2 plugin (Callahan et al., 2016). Microbial community analyses were performed in R (version 4.1.2) using the microeco package (Edgar, 2013). The relative abundances of the top 10 phyla and genera were compared to illustrate shifts in dominant taxa following treatment. Linear discriminant analysis effect size (LEfSe) was used to identify group-specific biomarkers (Segata et al., 2011), with a logarithmic LDA score threshold of 3.0 and *p* < 0.05.

### Co-occurrence network construction

Co-occurrence networks were constructed using CoNet (Faust and Raes, 2016) within Cytoscape. Features occurring at least once with valid sample counts >60% (minimum row sum = 0.01) were retained. A Spearman’s correlation threshold of 0.9 (*p* < 0.05) was applied to identify microbial interactions. Hub nodes were determined using the Matthews correlation coefficient (MCC) (Zhu, 2020), and the top 20 hub nodes were extracted for sub-network analysis. Network properties were calculated in Cytoscape, with node size proportional to degree (number of connections). Modules were identified using the MCODE plugin (Bader and Hogue, 2003) with parameters set as degree cutoff > 2, node score > 0.2, K-core = 2, and maximum depth = 100. Modules with a score > 4 were visualized. Network visualization was conducted using Gephi (Jacomy et al., 2014).

### Statistical analysis

Physiological and colonic transit characteristics were compared across groups using one-way analysis of variance (ANOVA) followed by Duncan’s test. Data are expressed as mean ± standard deviation (SD), with statistical significance set at *p* < 0.05. Heatmaps of standardized bacterial relative abundances were generated with the pheatmap package (v. 1.0.12) (Kolde, 2015). Correlations between physiological/colonic indices and microbial community composition were assessed using the Mantel test (Spearman correlation, *p* < 0.05) via the linkET R package (v. 0.0.7.4). Random forest classification (2,000 trees) was performed using the randomForest package (v. 4.7-1.1) (Svetnik et al., 2003) to identify taxa associated with the DI. All figures in the manuscript were generated using Adobe Illustrator CC 2019.

## Results

### Physiological and colonic characteristics of rats

Post-intervention analyses revealed significant differences (*p* < 0.05) in key physiological and colonic parameters across groups (Table 1). The body mass of IBS-D rats in the M, Ac, Mx, and P groups decreased by 12.7, 7.4, 6.3, and 8.7%, respectively, compared with the control group (C: 378 ± 5 g; *p* < 0.05). BSFS scores for the C, M, Ac, Mx, and P groups were 4, 6, 4, 4, and 5, respectively. Type 4 stools (smooth and soft, normal consistency) were predominant in C, Ac, and Mx, whereas rats in M exhibited type 6 stools (fluffy pieces with mushy consistency), confirming diarrheal pathology. Rats in P partially alleviated symptoms, as indicated by type 5 stools (soft blobs), although incomplete recovery persisted.

**Table 1:** Comparisons of the measured Physiological characteristics between five groups.

Categories	C	M	Ac	Mx	P
Mass (g)	378 ± 5a	330 ± 2d	350 ± 4b	354 ± 6b	345 ± 4c
BSFS	4 ± 1c	6 ± 1a	4 ± 1bc	4 ± 1bc	5 ± 1b
DI	0 ± 0c	1.10 ± 0.29a	0.44 ± 0.08b	0.32 ± 0.17b	0.48 ± 0.17b
FWC (%)	44 ± 4c	60 ± 4a	46 ± 4c	45 ± 4c	51 ± 4b
FP	3 ± 1d	5 ± 1a	4 ± 1bc	3 ± 1 cd	5 ± 1ab
CTT (min)	117 ± 52a	48 ± 17b	132 ± 20a	108 ± 37ab	83 ± 16b

Numbers are given as the average ± standard deviation. Different lowercase letters (a, b, c, d) indicate significant differences among groups (Duncan, *p* < 0.05). Mass: body mass of rats, BSFS, Bristol Stool Form Scale score, DI, Diarrhea index, FWC, Fecal Water Content, FP, Fecal Pellet counts, CTT, Colonic Transit Time.

DI was markedly reduced (*p* < 0.05) from 1.10 in M to 0.44 (Ac), 0.32 (Mx), and 0.48 (P), indicating therapeutic efficacy across all interventions. FWC in M (60%) and P (51%) remained significantly higher than C (44%, *p* < 0.05), while Ac (46%) and Mx (45%) maintained levels comparable to healthy controls. The number of FP was 3 in C, 5 in M, 4 in Ac, 3 in Mx, and 5 in P. Compared with C, FP was significantly elevated in the M, Ac, and P groups (*p* < 0.05), whereas Mx exhibited no significant difference, highlighting its regulatory effect on gastrointestinal motility in IBS-D rats. CTT was 117 min in C and was markedly reduced in M (59.0% reduction, *p* < 0.05) and P (29.1% reduction, *p* < 0.05). In contrast, Mx induced only a modest reduction (7.7%), whereas Ac prolonged CTT by 12.8%, suggesting potentially distinct mechanisms of action between acupuncture and moxibustion.

### Biomarker identification of gut microbiota across interventions

**Figure 1:**
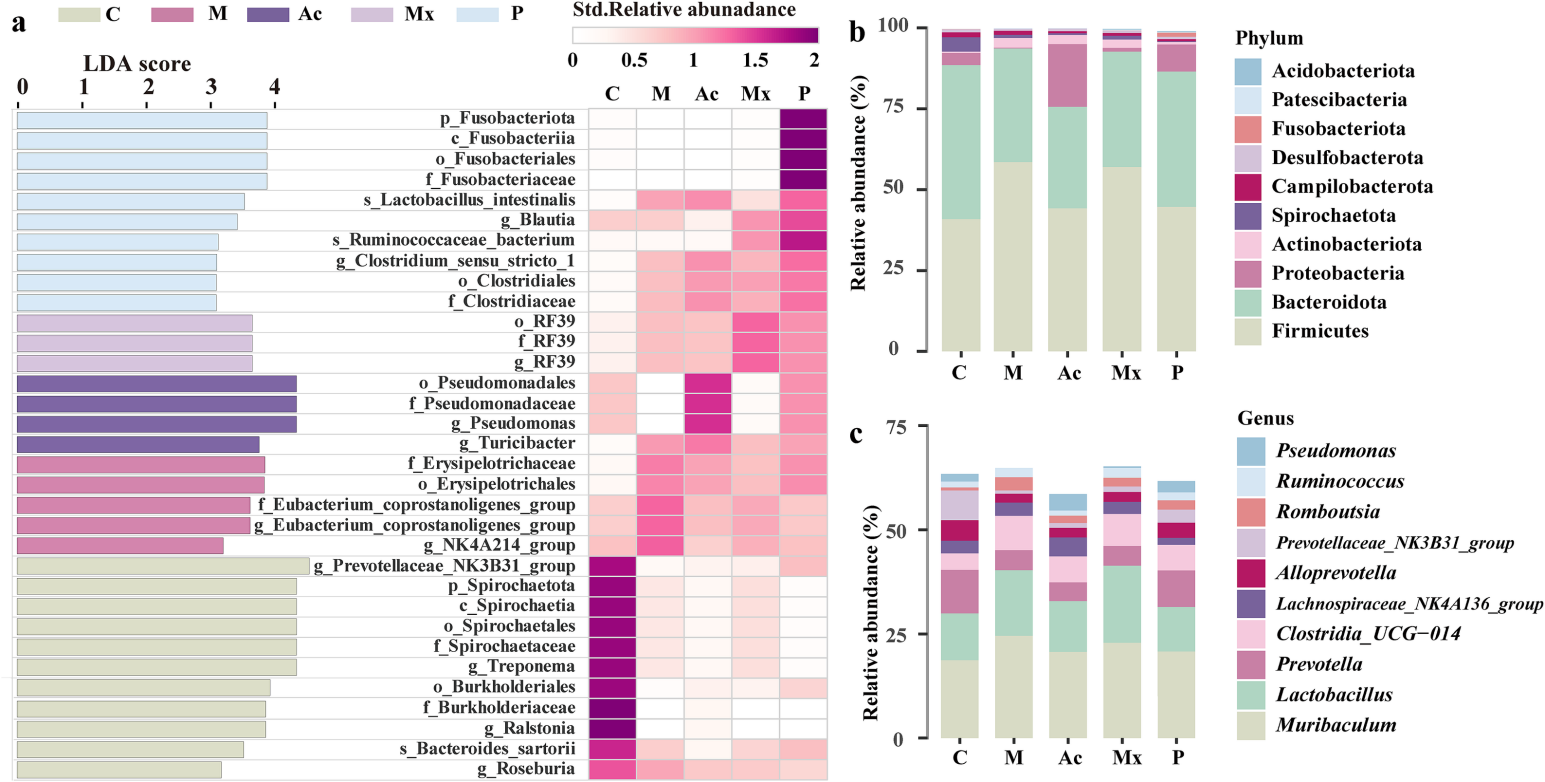
The biomarkers and distribution traits in five groups. **(a)** The bacterial taxa identified as biomarkers in five groups by LEfSe (LDA>3, *p* < 0.05) (left), and the Heatmap based on the standardized relative abundance (right). The darker the color, the higher the relative abundance of those biomarkers. **(b)** The relative abundance of the top 10 bacterial phyla in five groups. **(c)** The relative abundance of the top 10 bacterial genera in five groups.

A total of 33 microbial taxa were identified as discriminative biomarkers (LDA > 3, *p* < 0.05) across the five experimental groups (Figure 1a), revealing distinct gut microbial signatures among healthy rats, IBS-D model rats, and those receiving different interventions. Eleven biomarkers were enriched in Group C, distinguishing healthy rats from IBS-D rats. Among them, Spirochaete (relative abundance: 4.5%) was the sole phylum-level biomarker and ranked among the top 10 in healthy rats (Figures 1a,b). Four genera (*Prevotellaceae_NK3B31_group*, *Ralstonia*, *Roseburia*, and *Treponema*) were recognized as biomarkers in Group C. *Prevotellaceae_NK3B31_group*, the dominant genus in the gut microbiota of rats, exhibited the highest relative abundance in C (7.1%) among all groups (Figures 1a,c). Five taxa were identified as biomarkers in M, including the genera of *Eubacterium_coprostanoligenes_group* (1.5%) and *Ruminococcaceae_NK4A214_group* (0.6%). These two genera had higher relative abundance in M than the other four groups, suggesting a potential association with microbial imbalance and IBS-D pathogenesis (Figure 1a). Four taxa were enriched in Group Ac, including *Pseudomonas* (4.0%) and *Turicibacter* (1.2%), both of which were significantly more abundant in this group than in others (Figure 1a). These taxa consistently showed stratified enrichment across all replicates, supporting their role as potential biomarkers of acupuncture’s therapeutic effects in IBS-D rats. Among the three biomarkers identified in group Mx, the genus *RF39* (1.1%) had the highest abundance, indicating its potential role as a key genus regulated by moxibustion in IBS-D rats (Figure 1a). Group P had 10 biomarkers, including the phylum of Fusobacteriota (1.2%) and the genera of *Blautia* (0.6%) and *Clostridium_sensu_stricto_1* (0.3%). Their enrichment highlights a pharmacotherapy-specific microbial regulation pattern.

At the phylum level, the dominant taxa across groups were Firmicutes (40.9%–59.0%), Bacteroidota (31.4%–47.6%), and Proteobacteria (0.24%–19.4%). The Firmicutes proportion was highest in the M group (58.5%) and lowest in the Ac group (44.2%), whereas Bacteroidota was highest in the C group (47.6%) and lowest in the M group (35.2%). The Proteobacteria proportion increased notably in the Ac group (19.4%) compared to other groups (ranging from 0.24% to 3.75%). At the genus level, the three most abundant genera were *Muribaculum* (18.7%–24.5%), *Lactobacillus* (10.7%–18.5%), and *Prevotella* (4.5%–10.5%). *Muribaculum* was most abundant in the M group (24.5%), *Lactobacillus* was highest in the Ac group (18.5%), and *Prevotella* was most abundant in the C group (10.5%).

### Community-level interactions of gut microbiota

**Figure 2:**
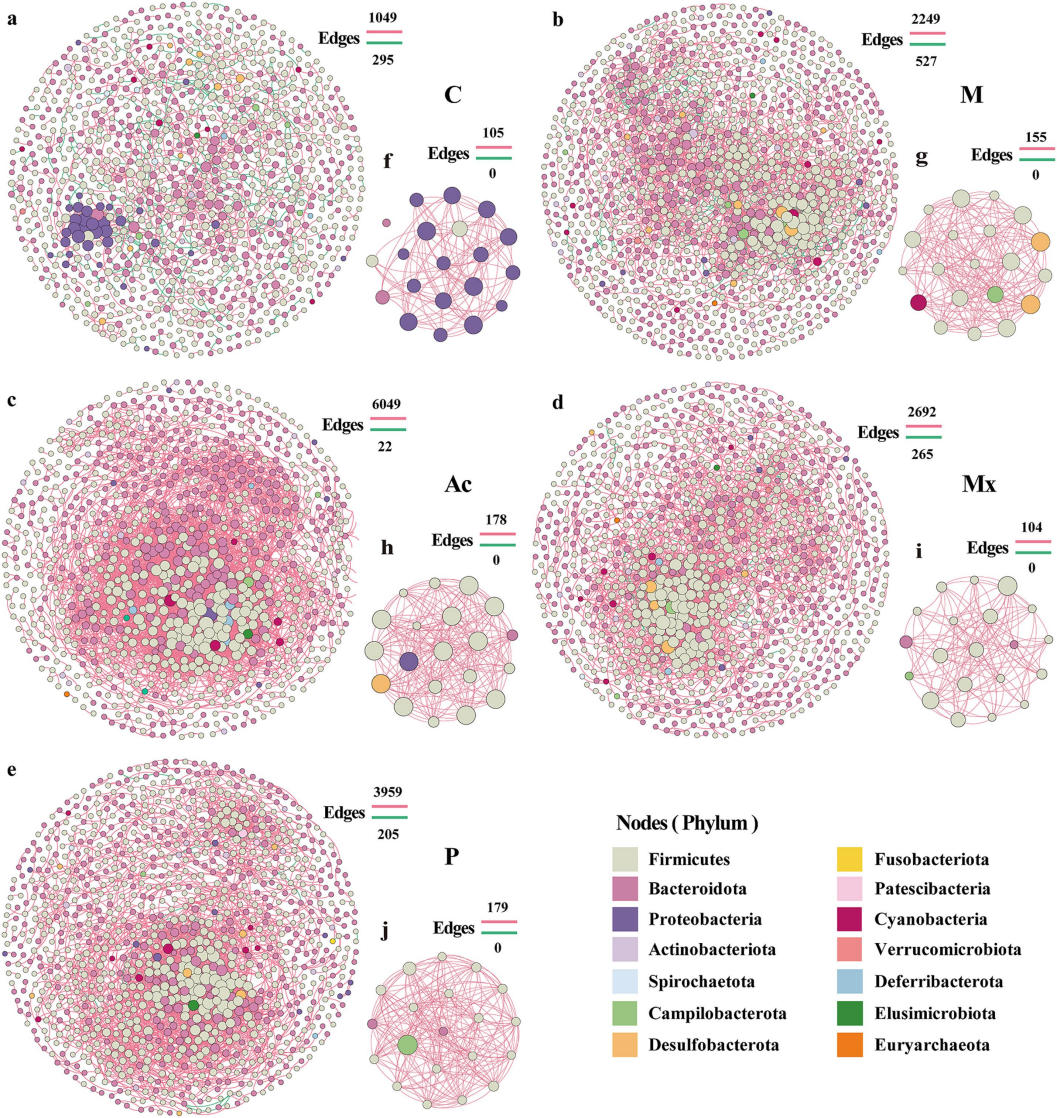
Network patterns of rat gut microbe communities. Networks of **(a)** C, **(b)** M, **(c)** Ac, **(d)** Mx, and **(e)** P treated groups. The hub networks (between the top 20 key taxa) of **(f)** C, **(g)** M, **(h)** Ac, **(i)** Mx, and **(j)** P were based on their networks. A connection indicates a strong (Spearman’s |r| > 0.95) and significant (p < 0.05) correlation. Positive correlations are represented by red lines, whereas negative correlations are represented by green lines. The size of the nodes corresponds to the degree of operational taxonomic units (OTUs).

To elucidate interspecies dynamics within the gut microbiota, network analysis was performed on taxon abundance profiles across five intervention groups. The number of edges ranged from 1,344 ~ 6,071, and the number of nodes from 934 ~ 1,159 across five groups (Figure 2; Supplementary Table S2). Compared with Group C (983 nodes, 1,344 edges), Group M and the three treatment groups (Ac, Mx, P) exhibited variable increases in nodes and edges, except for a reduction in node number in Ac. These changes indicated an overall increase in the network complexity of gut microbiota in IBS-D rats (Figure 2; Supplementary Table S2). The highest node number (1,159) and the largest number of negative correlations (527) in group M suggested a potential microbiota imbalance in IBS-D rats (Figure 2b; Supplementary Table S2). In the Ac group, the number of nodes in the network was the lowest, but the number of edges reached as high as 6,071, including 99.6% of edges with positive correlations. The enhanced positive interactions within gut microbiota may be associated with the therapeutic effects of acupuncture in IBS-D rats (Figure 2c; Supplementary Table S2).

The Mx group exhibited the largest network radius (13), indicating a relatively loose structure and scattered node distribution (Figure 2d; Supplementary Table S2). In contrast, the P group showed the highest heterogeneity (1.127), with a few highly connected nodes coexisting with many poorly connected nodes, reflecting an unbalanced connectivity pattern (Figure 2e; Supplementary Table S2). Compared with Group C (network diameter: 34; characteristic path length: 12.851), the other four groups generally showed reduced diameters (21–24) and shorter path lengths (6.069–7.284). Meanwhile, the average number of neighbors (e.g., 14.763 in Ac vs. 3.598 in C), clustering coefficient (0.403 in Ac vs. 0.288 in C), density (0.018 in Ac vs. 0.007 in C), and centralization (0.076 in Ac vs. 0.030 in C) were markedly increased, with the highest values observed in the Ac group (Supplementary Table S1). These findings suggested that network connectivity was strengthened, and central nodes with strong local clustering emerged in treatment groups, particularly under acupuncture intervention.

The nodes of the hub network of the C group mainly belonged to Proteobacteria (Figure 2f), but in the other four groups, Firmicutes were the most abundant phylum (Figures 2g–j), which indicated that Firmicutes were the key taxa associated with the attack and development of IBS-D diseases. The absence of Bacteroidota in the hub network of M suggested that this phylum was essential for maintaining gut microbiota homeostasis. Desulfobacterota appeared in the hub network of M and Ac, and Campilobacterota occurred in M, Mx, and P. Cyanobacteria were the unique phylum that occurred in M (Figure 2; Supplementary Table S2). Although these phyla comprised only 15–20% of the proportion in the hub network, they may contribute to the treatment differences of IBS-D. Notably, several genera that emerged as hub nodes also overlapped with the most abundant taxa, such as *Prevotella* (Ac), *Muribaculum* (Mx), and *Blautia* (P). Their presence in both the hub networks and as dominant community members highlighted their central role in shaping microbial interactions under IBS-D conditions and distinct interventions.

### Network-level alterations of bacterial co-occurrence under interventions

**Figure 3:**
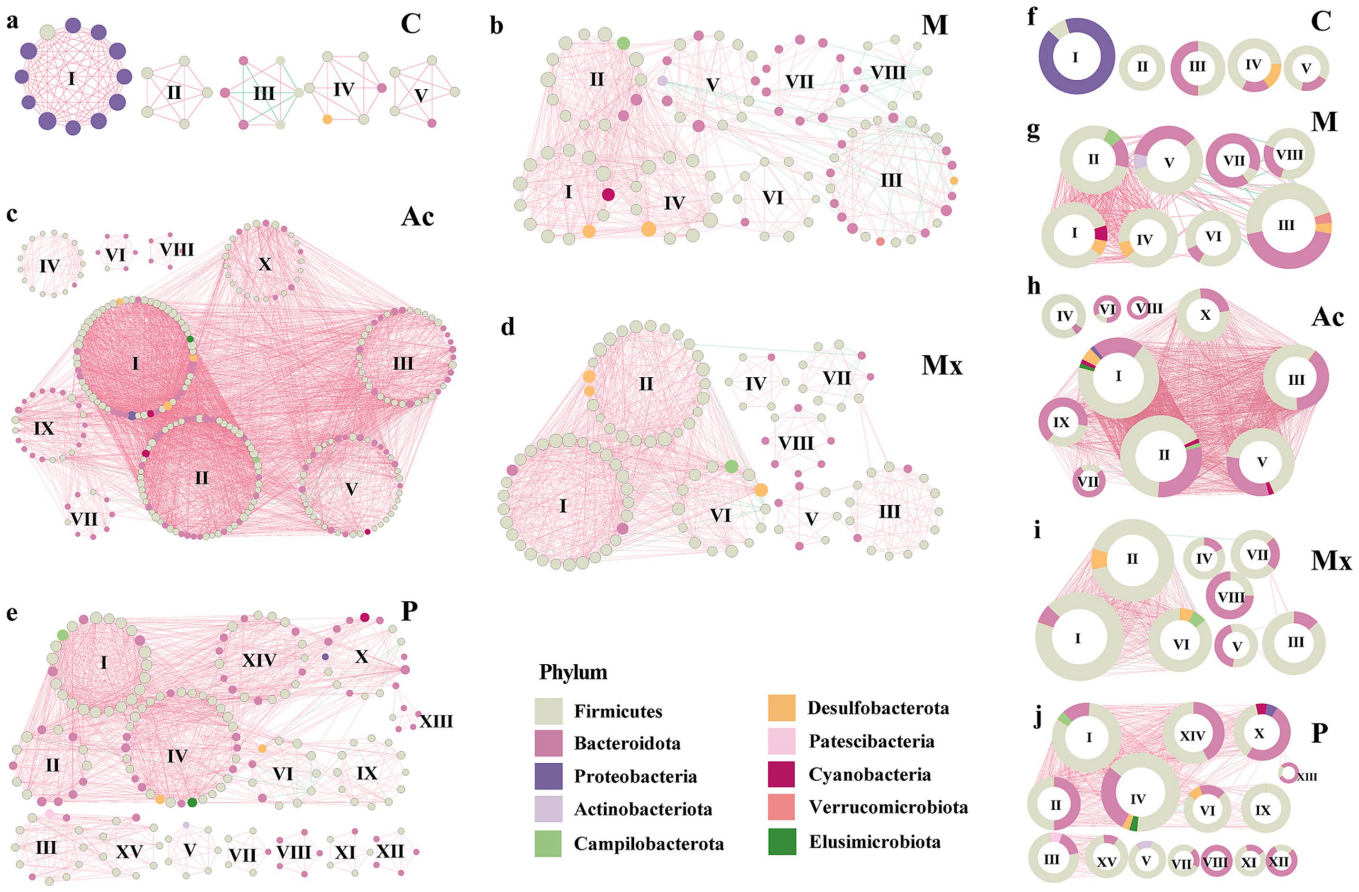
Modularity of bacterial networks in the gut of rats under five specific interventions. A co-occurrence in the gut of rats of healthy controls **(a)** C, IBS-D model **(b)** M, Acupuncture treated **(c)** Ac, Moxibustion **(d)** Mx, and Pharmacotherapy **(e)** P groups. Colors of nodes indicate the different major phyla. The hub networks (between the top 20 key taxa) of **(f)** C, (g) M, **(h)** Ac, **(i)** Mx, and **(j)** P. A connection indicates a strong (Spearman’s |r| > 0.9) and significant (*p* < 0.05) correlation. Positive correlations are represented by red lines, whereas negative correlations are represented by green lines. The size of the nodes corresponds to the degree of OTUs. The pie charts represent the composition of modules with the modularity scoring >4 in the co-occurrence network.

The modularity patterns of co-occurrence networks revealed distinct community organizations under different interventions. Across the five groups (C, M, Ac, Mx, and P), we identified 5, 8, 9, 8, and 15 modules (modular score > 4), respectively (Figure 3). In group C, the five modules represented clusters of OTUs that were highly interconnected within modules but lacked connections to OTUs outside the clusters (Figures 3a,f). In contrast, the other four groups exhibited extensive inter-module connections, suggesting that the stability of gut microbial communities was influenced by cross-cluster interactions, which may be regulated by the three interventions to alleviate IBS-D symptoms. Besides, compared with group C (five modules), the Ac group exhibited more modules with larger average sizes, whereas the P group showed the highest number of smaller modules (15), suggesting more fragmented but diverse microbial restructuring under pharmacotherapy.

Module I in group C was composed primarily of Proteobacteria (91.7%) and Firmicutes (8.3%), with a modularity score of 11.1. Modules II–V were dominated by Firmicutes (50%–100%), and Bacteroidota was 0%–50%. They were in modules II, III, IV, and V of C, with the modularity scoring 4.5–5.0. Notably, negative correlations were observed in Module III, where Firmicutes and Bacteroidota each accounted for 50% of the composition (Figures 3a,f). These results suggested that the dominance of Proteobacteria, together with the balance between Firmicutes and Bacteroidota, was critical for maintaining microbial network stability in healthy rats.

In group M, Verrucomicrobiota (4.0%) appeared uniquely in module III, co-occurring with Firmicutes, Bacteroidota, and Desulfobacterota, which suggested a potential role in triggering IBS-D pathology (Figures 3b,g). The consistent detection of Campilobacterota across intervention groups (1.7%–7.1%) suggested that this phylum might act as a persistent microbial signature associated with IBS-D, potentially influencing treatment responsiveness. Actinobacteriota were enriched in Module V of M (7.1%) and Module V of P (14.3%). Except for the C and Mx groups, Cyanobacteria and Elusimicrobiota also appeared (1.7%–7.7%). Collectively, these findings indicated that the effects of interventions were strongly dependent on the heterogeneity and taxonomic composition of network modules.

### Associations between key bacterial taxa and host physiological characteristics

**Figure 4:**
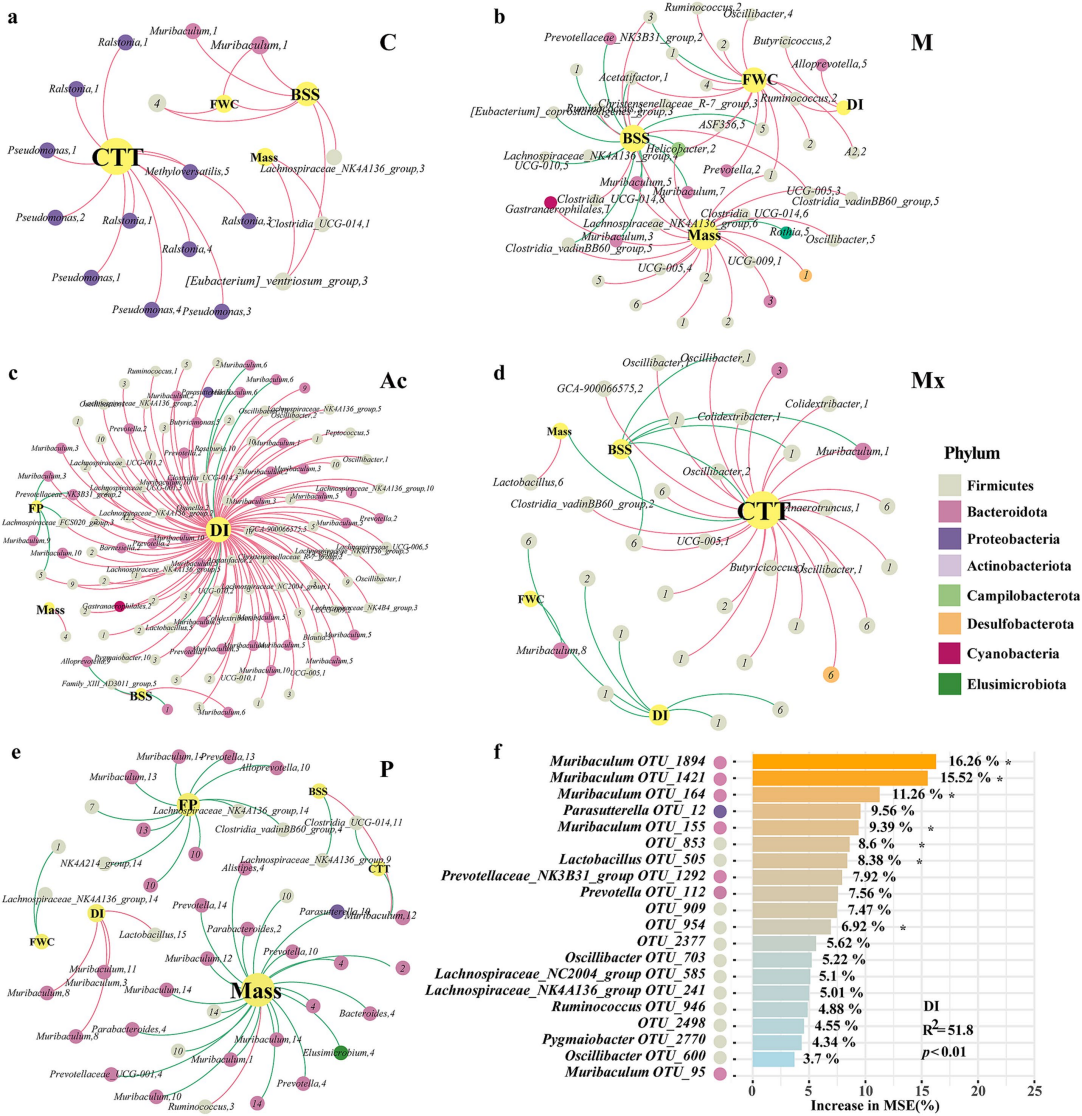
Relevance of key taxa associated with IBS-D in rats under different interventions. The co-occurrence networks of the physiological parameters and key taxa in the gut of **(a)** healthy controls C, **(b)** IBS-D model (M), **(c)** Acupuncture (Ac), **(d)** Moxibustion (Mx), and **(e)** Pharmacotherapy (P) rats. Colors of nodes indicated the different major phyla. The node size represents the degree of the OTUs. The red links represent positive correlations (*p* < 0.05), while green links represent negative correlations (*p* < 0.05). **(f)** The contribution of key taxa to DI, analyzed by random forest, *represented the significant contribution.

A total of 538 OTUs identified as network modular nodes were selected as key taxa for subsequent analyses. We constructed co-occurrence networks between these taxa and host physiological parameters under the five interventions. Marked differences among the networks reflected distinct therapeutic mechanisms of acupuncture, moxibustion, and pharmacotherapy in IBS-D rats. In general, the co-occurrence networks of C and Mx groups were mainly driven by CTT, and their structures were simpler compared with those of the other groups. Negative correlations between DI and the phyla of Bacteroidota and Firmicutes were observed in the Ac and Mx groups, suggesting that these taxa may have been modulated by the interventions to alleviate diarrhea (Figure 4).

In group C, CTT, FWC, BSFS, and Mass represented the largest nodes, with BSFS showing a positive association with FWC. Genera such as *Pseudomonas*, *Ralstonia*, and *Methyloversatilis*, which belonged to Proteobacteria, were positively correlated with CTT (Figure 4a). In the M group, the network was driven by BSFS, Mass, FWC, and DI. *Butyricicoccus* and *Ruminococcus* (Firmicutes, module II) and *Alloprevotella* (Bacteroidota, module V) were positively associated with DI, whereas *Muribaculum* (Bacteroidota; modules III, V, and VII) and seven other genera were negatively associated with BSFS (Figure 4b). In the Ac group, DI was the dominant node, linked with 28.1% of Bacteroidota and 62.0% of Firmicutes. *Muribaculum* (module VI) and *Lactobacillus* (Firmicutes, module V) showed negative associations with DI. *Alloprevotella* (module IX) was negatively associated with BSFS, *Muribaculum* (modules III, XI), and two additional genera were negatively associated with FP (Figure 4c). In the Mx group, CTT, DI, BSFS, FWC, and Mass were the largest nodes. CTT was negatively correlated with BSFS and Mass. Firmicutes was the main phylum driven by DI, BSFS, and CTT; its correlations with DI or BSFS were negative, but positive with CTT. *Muribaculum* (module I) showed positive associations with CTT, whereas *Muribaculum* (module VIII) was negatively associated with FWC (Figure 4d). In the P group, Bacteroidota dominated the co-occurrence network. This phylum negatively influenced mass, FP, CTT, BSFS, and FWC, whereas DI showed the opposite pattern. Interestingly, *Muribaculum* (modular III, VII) and Lactobacillus (modular XV) were positively correlated with DI (Figure 4f). These results indicated that Firmicutes and Bacteroidota, as well as their relative ratio, together with intra-module interactions and specific key genera such as *Muribaculum*, were vital in regulating the microbial ecosystem.

The random forest model explained 47.5% of the variance in DI, and the full model was significant (R^2^ = 51.8, *p* < 0.01). Top contributing OTUs were OTU_1894, OTU_1,421, OTU_164, and OTU_155 (all assigned to *Muribaculum*) and OTU_505 (*Lactobacillus*), each showing significant (*p* < 0.05) variable importance (Figure 4f). Group-wise standardized abundances (Supplementary Figure S1) showed that *Muribaculum* OTU_1894, OTU_1,421, and OTU_155, as well as *Lactobacillus* OTU_505, were highest in M, whereas *Muribaculum* OTU_164 was highest in C. Overall, the DI-associated taxa predominantly belonged to Firmicutes and Bacteroidota. Across groups, *Muribaculum* showed negative associations with DI in Ac, positive associations with CTT, negative associations with FWC in Mx, and positive associations with DI in P (Figures 4a–e). Group-dependent differences were also observed in the network neighbors and module membership of *Muribaculum* and *Lactobacillus*. These results indicated that the intervention groups differed in how these taxa were positioned within the microbial networks.

## Discussion

This study compared acupuncture, moxibustion, and pharmacotherapy in a validated IBS-D rat model and linked symptom readouts with 16S rRNA gene sequence, biomarker discovery, and co-occurrence network analyses. Acupuncture and moxibustion alleviated diarrhea symptoms more than pinaverium bromide, and each intervention showed a distinct pattern of community structure and group-specific taxon–phenotype associations. A total of 33 microbial taxa were identified as biomarkers, which were associated with different interventions on rats. Further, we found that the ratio of Firmicutes to Bacteroidetes was crucial to the attack of IBS-D, and certain key genera (e.g., *Muribaculum*) within the network can be regulated by different therapeutic interventions to alleviate the symptoms of rats with IBS-D.

Current evidence supports the therapeutic potential of acupuncture and moxibustion for IBS-D, and our findings were consistent with this pattern. Prior studies have shown that IBS-D symptoms are reduced with acupuncture and superior stool normalization with moxibustion compared with oral medications (Bao et al., 2016; Dai et al., 2022; Guo et al., 2022), and other researchers suggested that acupuncture alleviates diarrheal symptoms with efficacy greater than pharmacotherapy (Pei et al., 2020; Sun et al., 2011). In the present study, acupuncture and moxibustion improved multiple diarrhea-related values, including fecal consistency (BSFS), fecal water content (FWC), fecal pellet counts (FP), and colonic transit time (CTT) (*p* < 0.05), with effects exceeding those observed with pharmacotherapy (Table 1). Notably, moxibustion was particularly effective in reducing fecal pellet counts (*p* < 0.05) (Table 1). These findings supported greater amelioration of IBS-D-related stool and transit abnormalities with acupuncture and moxibustion than with pharmacotherapy in this model.

Group-specific biomarkers suggested divergent mechanisms of action. In the C group, *Prevotellaceae_NK3B31_group* was a biomarker and a dominant genus in the healthy rat gut microbiota (relative abundance 7.1%), supporting its potential role in maintaining intestinal homeostasis (Figure 1). In the co-occurrence networks of M and Ac, the relative abundance of *Prevotellaceae_NK3B31_group* (Bacteroidota) was negatively correlated with BSFS, DI, and FP, which was consistent with the interpretation that acupuncture was associated with improved stool form, lower diarrhea index, and fewer pellets via changes involving this genus (Figures 4b,c).

*Muribaculum* (Bacteroidota) was a highly abundant genus. In the C network, *Muribaculum* within module I showed a positive correlation with BSFS and a negative correlation with DI. In the Ac network, *Muribaculum* in module VI showed a negative correlation with DI, and in modules III and XI, it was negatively correlated with FP (Figure 4c). In the Mx network, *Muribaculum* in module VIII was negatively correlated with DI, whereas *Muribaculum* in modules I and III was positively correlated with CTT (Figure 4d). In the P network, *Muribaculum* in modules III, VIII, and XI was negatively correlated with DI (Figure 4f). These patterns indicated that moxibustion was associated with altered interactions between *Muribaculum* and other taxa, accompanied by symptom profiles approaching those of controls. By contrast, the P network was characterized by predominantly negative bacteria and physiological parameters correlations (Figure 4). Biomarker abundances differed across the five groups and served as discriminative features. Because specific taxa are often linked to particular functions, the biomarker sets likely reflected group-wise differences in intestinal function and in the organization of microbial interaction networks under the different interventions.

Network and module analyses of the gut microbiota revealed key community features under different interventions. In the C group, module I was primarily composed of Proteobacteria (91.7%) and Firmicutes (8.3%), with a modularity score of 11.1. Modules II–V were dominated by Firmicutes (50%–100%), with Bacteroidota ranging from 0%–50%. These patterns indicated that the healthy gut microbiota exhibited well-structured modular organization with distinct phylum-level compositions. In comparison, the M group showed the largest number of nodes, suggesting the emergence of a greater number of interacting taxa in IBS-D (Figure 2; Supplementary Table S2). Notably, the co-occurrence network of the Mx group closely resembled that of the C group, implying that moxibustion preserved a more control-like organization of gut microbial communities. Consistent with previous studies, Jalanka-Tuovinen et al. (2014) reported a 12-fold expansion of Bacteroidota in IBS patients, which aligns with our observation that Bacteroidota contributed substantially to several modules in the IBS-D model group.

Acupuncture and moxibustion demonstrated superior effects over pharmacotherapy in alleviating IBS-D symptoms, by better normalizing stool form, diarrhea index, and fecal water content. Biomarker profiling revealed unique microbial signatures for each intervention, with acupuncture enriching *Pseudomonas* and *Turicibacter* and moxibustion enhancing RF39. Network analysis revealed that acupuncture fosters tightly connected microbial networks, moxibustion maintains control-like structure, and pharmacotherapy displays fragmented connectivity. Taxon-physiological parameter associations varied by treatment, with acupuncture and moxibustion more effectively regulating key taxa like *Muribaculum* to alleviate diarrhea.

However, our study has several limitations. The pathogenesis of IBS-D is complex, involving inflammatory responses and multiple pathways, with significant variations in symptoms, patient constitutions, and underlying causes. A deeper understanding of the pathophysiological mechanisms of IBS-D is needed, incorporating factors such as genomic characteristics, gut microbiota composition, and other clinical indicators. Further studies are needed to explore the effects of acupuncture and moxibustion on IBS-D patients, particularly focusing on the mechanisms driving changes in immune responses and gut-brain axis signaling.

## Conclusion

In this study, we compared the efficacy of acupuncture, moxibustion, and pharmacotherapy in treating IBS-D and their effects on gut microbiota. Analysis of physiological and colonic characteristics revealed that acupuncture and moxibustion demonstrated superior therapeutic effects over pharmacotherapy in alleviating IBS-D symptoms. A total of 33 microbial taxa were identified as biomarkers, showing distinct associations with the different interventions. Additionally, we found that the ratio of Firmicutes to Bacteroidetes played a crucial role in IBS-D pathogenesis, and specific genera, such as *Muribaculum*, were modulated by the therapeutic interventions to help alleviate symptoms in IBS-D rats.

## Data Availability

The datasets presented in this study are publicly available. This data can be found at: https://www.ncbi.nlm.nih.gov/sra, accession number PRJNA1226893.
