# Supplementary material for: Comparison of acupuncture, moxibustion, and pharmacotherapy in improving diarrhea-predominant irritable bowel syndrome
Source: Front Microbiol. 2025 Oct 2;16:1638930. doi: 10.3389/fmicb.2025.1638930 (PMC12528028; doi:10.3389/fmicb.2025.1638930)
Supplement: Supplementary file 1 [file Data_Sheet_1.docx]

**Supplementary Information**

**Comparison of acupuncture, moxibustion, and pharmacotherapy in improving diarrhea-predominant irritable bowel syndrome**

Peiqin Zhang ^1, 2 †^, Yao Chen ^1 †^, Biyu Lai ^1^, Shuangshuang Wang ^1^, Dan Li ^1^, Runlin Wen ^1^, Yaping Duan ^1^, Dan Liu ^1^, Bo Li ^1 #^, Chang She ^1 #^

*^1^ Changsha Hospital of Traditional Chinese Medicine (Changsha Eighth Hospital), Changsha, China*

*^2^ Institute of Rehabilitation and Health Care, Department of Rehabilitation and Traditional Chinese Medicine, Hunan Traditional Chinese Medical College, Zhu zhou,China.*

^*^ These authors contributed to the work equally and should be regarded as co-first authors.

^#^ Corresponding author: Bo Li, Chang She

*E-mail address*: [shechang159@163.com](mailto:shechang159@163.com)

**
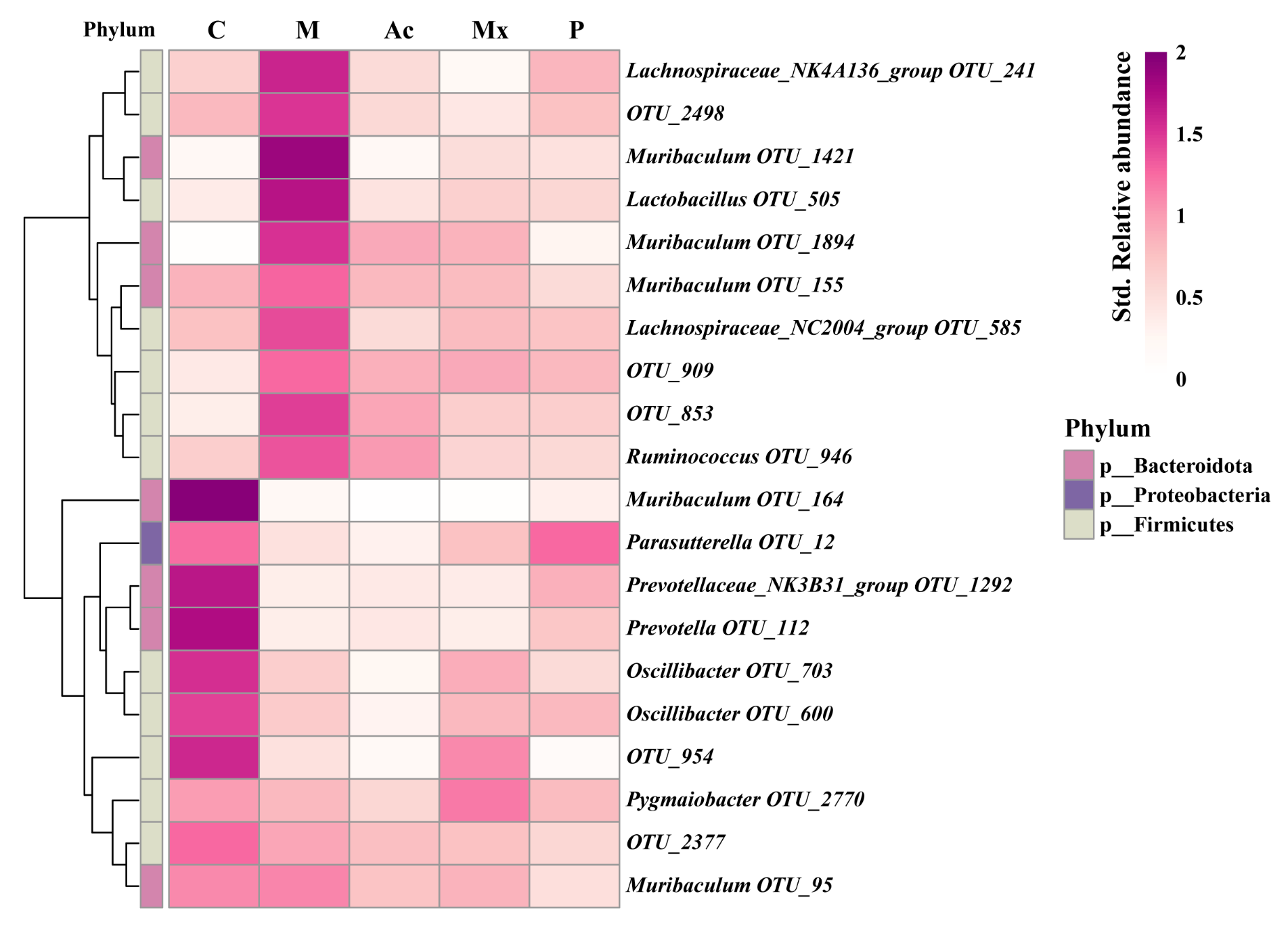
**

**Figure S1. Connections between bacterial network modulars and physiological properties in different groups.** Pairwise Spearman’s correlation matrix of the physical properties of mice was shown with block charts. and bacterial network modulars were related to the soil properties by Mantel tests. Edge width means the Mantel’s statistic, and edge color means the statistical significance.

**Table S1**. Topological properties of gut microbe networks in five intervention groups.

| Network Indexes | C | M | Ac | Mx | P |
| --- | --- | --- | --- | --- | --- |
| Number of nodes | 983 | 1159 | 934 | 1001 | 1085 |
| Number of edges | 1344 | 2776 | 6071 | 2957 | 4164 |
| Avg. number of neighbors | 3.598 | 6.188 | 14.763 | 7.171 | 8.388 |
| Network diameter | 34 | 22 | 21 | 24 | 23 |
| Network radius | 11 | 12 | 11 | 13 | 12 |
| Characteristic path length | 12.851 | 6.799 | 6.069 | 7.268 | 7.284 |
| Clustering coefficient | 0.288 | 0.285 | 0.403 | 0.323 | 0.345 |
| Density | 0.007 | 0.008 | 0.018 | 0.009 | 0.009 |
| Heterogeneity | 0.784 | 1.005 | 1.038 | 1.121 | 1.127 |
| Centralization | 0.030 | 0.038 | 0.076 | 0.048 | 0.048 |
| Connected components | 112 | 119 | 42 | 70 | 44 |

**Table S2**. The information of hub network under five intervention groups.

| Group | ID | degree | Genus | Phylum |
| --- | --- | --- | --- | --- |
| C | OTU-302 | 7 |  | Proteobacteria |
|  | OTU-296 | 9 | *Ralstonia* | Proteobacteria |
|  | OTU-268 | 16 | *Pseudomonas* | Proteobacteria |
|  | OTU-30 | 10 | *Pseudomonas* | Proteobacteria |
|  | OTU-344 | 12 | *Methyloversatilis* | Proteobacteria |
|  | OTU-293 | 12 | *Ralstonia* | Proteobacteria |
|  | OTU-16 | 12 | *Ralstonia* | Proteobacteria |
|  | OTU-300 | 15 | *Ralstonia* | Proteobacteria |
|  | OTU-315 | 17 | *Ralstonia* | Proteobacteria |
|  | OTU-19 | 15 | *Methyloversatilis* | Proteobacteria |
|  | OTU-412 | 15 |  | Bacteroidota |
|  | OTU-292 | 17 | *Pseudomonas* | Proteobacteria |
|  | OTU-357 | 8 | *RF39* | Firmicutes |
|  | OTU-34 | 17 | *Pseudomonas* | Proteobacteria |
|  | OTU-1035 | 9 | *Prevotellaceae_NK3B31_group* | Bacteroidota |
|  | OTU-402 | 14 | *Lactobacillus* | Firmicutes |
|  | OTU-10 | 14 | *Pseudomonas* | Proteobacteria |
|  | OTU-259 | 19 | *Ralstonia* | Proteobacteria |
|  | OTU-33 | 15 | *Pseudomonas* | Proteobacteria |
|  | OTU-700 | 11 | *Ralstonia* | Proteobacteria |
| M | OTU-2534 | 22 | *Butyricicoccus* | Firmicutes |
|  | OTU-286 | 22 | *UCG-009* | Firmicutes |
|  | OTU-972 | 28 | *Helicobacter* | Campilobacterota |
|  | OTU-3073 | 35 |  | Firmicutes |
|  | OTU-911 | 32 | *Ruminococcus* | Firmicutes |
|  | OTU-1069 | 30 |  | Firmicutes |
|  | OTU-576 | 24 |  | Firmicutes |
|  | OTU-2770 | 35 | *Pygmaiobacter* | Firmicutes |
|  | OTU-2777 | 27 | *Acetatifactor* | Firmicutes |
|  | OTU-957 | 22 |  | Firmicutes |
|  | OTU-242 | 28 |  | Firmicutes |
|  | OTU-21 | 29 |  | Desulfobacterota |
|  | OTU-629 | 21 |  | Firmicutes |
|  | OTU-1133 | 27 | *Gastranaerophilales* | Cyanobacteria |
|  | OTU-1031 | 31 | *[Eubacterium]_siraeum_group* | Firmicutes |
|  | OTU-925 | 35 |  | Firmicutes |
|  | OTU-590 | 33 |  | Firmicutes |
|  | OTU-585 | 32 | *Lachnospiraceae_NC2004_group* | Firmicutes |
|  | OTU-1043 | 27 |  | Firmicutes |
|  | OTU-7 | 37 |  | Desulfobacterota |
| Ac | OTU-945 | 64 |  | Firmicutes |
|  | OTU-77 | 59 | *Quinella* | Firmicutes |
|  | OTU-2534 | 53 | *Butyricicoccus* | Firmicutes |
|  | OTU-125 | 64 | *Prevotella* | Bacteroidota |
|  | OTU-12 | 74 | *Parasutterella* | Proteobacteria |
|  | OTU-2818 | 59 |  | Firmicutes |
|  | OTU-2873 | 48 |  | Firmicutes |
|  | OTU-2540 | 62 | *Christensenellaceae_R-7_group* | Firmicutes |
|  | OTU-513 | 72 | *Colidextribacter* | Firmicutes |
|  | OTU-7 | 67 |  | Desulfobacterota |
|  | OTU-879 | 58 |  | Firmicutes |
|  | OTU-2377 | 63 |  | Firmicutes |
|  | OTU-629 | 76 |  | Firmicutes |
|  | OTU-2378 | 76 | *Clostridia_vadinBB60_group* | Firmicutes |
|  | OTU-576 | 57 |  | Firmicutes |
|  | OTU-3073 | 60 |  | Firmicutes |
|  | OTU-286 | 64 | *UCG-009* | Firmicutes |
|  | OTU-249 | 61 | *UCG-010* | Firmicutes |
|  | OTU-2347 | 67 |  | Firmicutes |
|  | OTU-258 | 63 | *UCG-005* | Firmicutes |
| Mx | OTU-3059 | 34 |  | Firmicutes |
|  | OTU-345 | 26 | *Colidextribacter* | Firmicutes |
|  | OTU-972 | 40 | *Helicobacter* | Campilobacterota |
|  | OTU-1057 | 29 | *Incertae_Sedis* | Firmicutes |
|  | OTU-501 | 30 |  | Firmicutes |
|  | OTU-2377 | 27 |  | Firmicutes |
|  | OTU-909 | 44 |  | Firmicutes |
|  | OTU-1080 | 31 | *Ruminococcus* | Firmicutes |
|  | OTU-513 | 27 | *Colidextribacter* | Firmicutes |
|  | OTU-1304 | 30 | *Muribaculum* | Bacteroidota |
|  | OTU-321 | 41 | *Peptococcus* | Firmicutes |
|  | OTU-576 | 44 |  | Firmicutes |
|  | OTU-2534 | 35 | *Butyricicoccus* | Firmicutes |
|  | OTU-1112 | 24 |  | Firmicutes |
|  | OTU-1400 | 28 | *Bacteroides* | Bacteroidota |
|  | OTU-362 | 39 | *Clostridia_vadinBB60_group* | Firmicutes |
|  | OTU-581 | 31 |  | Firmicutes |
|  | OTU-955 | 28 | *Anaerotruncus* | Firmicutes |
|  | OTU-2385 | 30 |  | Firmicutes |
|  | OTU-398 | 28 |  | Firmicutes |
| P | OTU-374 | 52 |  | Firmicutes |
|  | OTU-247 | 46 |  | Firmicutes |
|  | OTU-106 | 37 |  | Bacteroidota |
|  | OTU-362 | 47 | *Clostridia_vadinBB60_group* | Firmicutes |
|  | OTU-3073 | 46 |  | Firmicutes |
|  | OTU-258 | 51 | *UCG-005* | Firmicutes |
|  | OTU-2306 | 35 |  | Firmicutes |
|  | OTU-2728 | 48 |  | Firmicutes |
|  | OTU-1166 | 40 |  | Firmicutes |
|  | OTU-2761 | 40 |  | Firmicutes |
|  | OTU-1150 | 42 | *Alloprevotella* | Bacteroidota |
|  | OTU-969 | 46 | *Ruminococcus* | Firmicutes |
|  | OTU-972 | 50 | *Helicobacter* | Campilobacterota |
|  | OTU-2550 | 45 |  | Firmicutes |
|  | OTU-3072 | 55 |  | Firmicutes |
|  | OTU-576 | 41 |  | Firmicutes |
|  | OTU-940 | 45 | *Blautia* | Firmicutes |
|  | OTU-941 | 48 | *Lachnospiraceae_UCG-006* | Firmicutes |
|  | OTU-740 | 45 | *Oscillibacter* | Firmicutes |
|  | OTU-585 | 46 | *Lachnospiraceae_NC2004_group* | Firmicutes |
